# Supplementary material for: Diagnostic Role of Four-Dimensional Computed Tomography for Preoperative Parathyroid Localization in Patients with Primary Hyperparathyroidism: A Systematic Review and Meta-Analysis
Source: Diagnostics (Basel). 2021 Apr 7;11(4):664. doi: 10.3390/diagnostics11040664 (PMC8068020; doi:10.3390/diagnostics11040664)
Supplement: Supplementary file 1 [file diagnostics-11-00664-s001.zip › Supplementary File/Supplementary Figures.pdf]

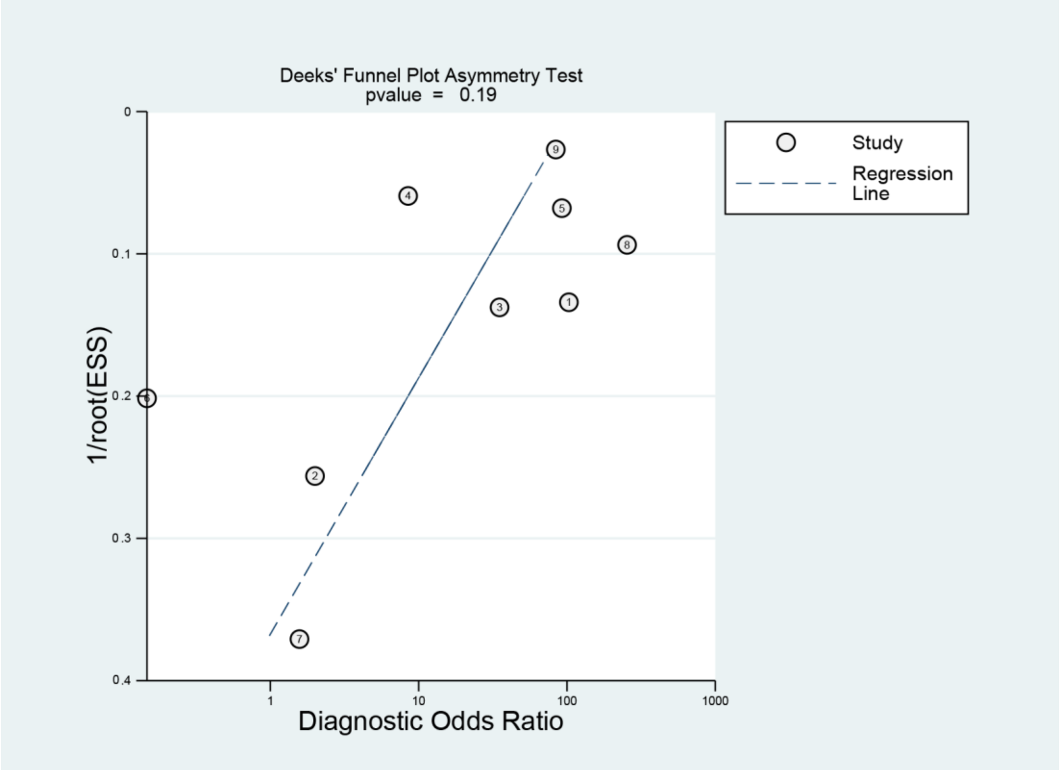


**Figure S1.** Deeks funnel plot asymmetry test for publication bias of 4D-CT for detecting HPG(s) on a per-lesion level.

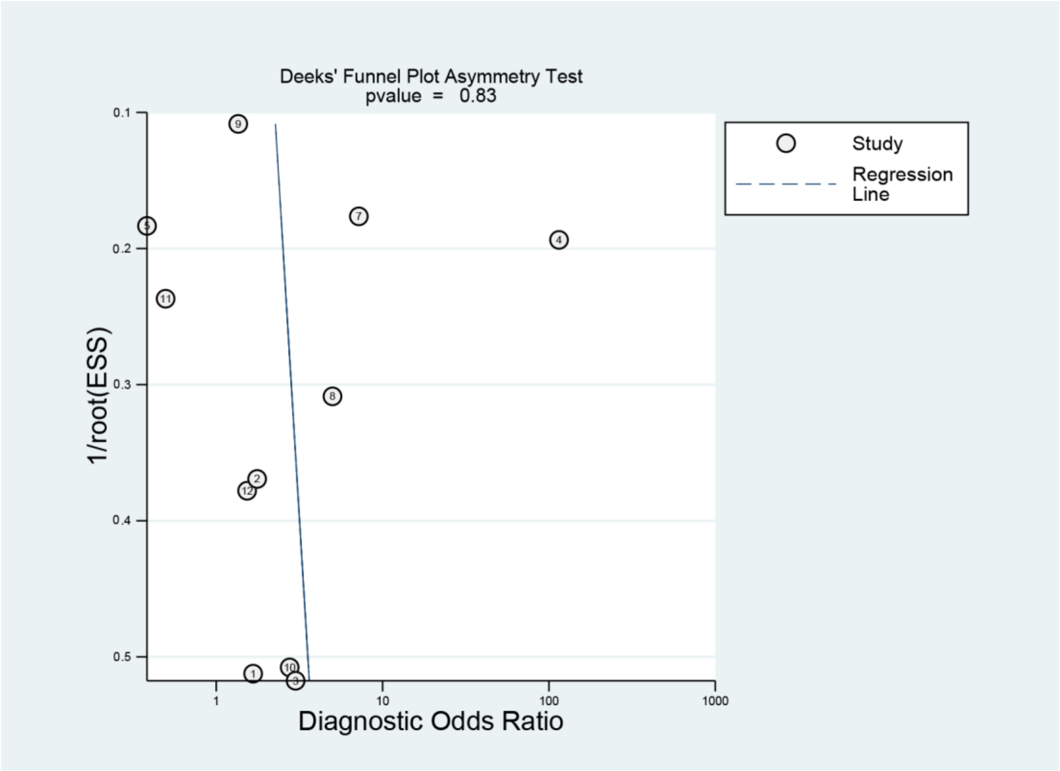


**Figure S2.** Deeks funnel plot asymmetry test for publication bias of 4D-CT for detecting HPG(s) on a per-patient level.
