# Supplementary material for: Diagnostic Role of Four-Dimensional Computed Tomography for Preoperative Parathyroid Localization in Patients with Primary Hyperparathyroidism: A Systematic Review and Meta-Analysis
Source: Diagnostics (Basel). 2021 Apr 7;11(4):664. doi: 10.3390/diagnostics11040664 (PMC8068020; doi:10.3390/diagnostics11040664)
Supplement: Supplementary file 1 [file diagnostics-11-00664-s001.zip › Supplementary File/Supplementary Files.pdf]

Search algorithm

Pubmed

“four-dimensional computed tomography” [MeSH] OR “4dct” [All Field] OR “4d-ct” [All Field] OR “four dimensional computed tomography” [All Field] AND “diagnostic accuracy” [All Field] OR “sensitivity” [All Field] OR “specificity” [All Field] AND “hyperparathyroidism” [All Field]OR “parathyroid adenoma” [All Field]

Embase

('four dimensional computed tomography'/exp OR 'four-dimensional computed tomography':ab,ti OR '4dct':ab,ti OR '4d-ct':ab,ti) AND ('hyperparathyroidism'/exp OR 'parathyroid adenoma'/exp) AND ('diagnostic accuracy'/exp OR 'sensitivity':ab,ti OR 'specificity':ab,ti)

Web of Science

1) TS=(“four-dimensional computed tomography” OR “4dct” OR “4d-ct” OR “four dimensional computed tomography”)
2) TS=(“diagnostic accuracy” OR “sensitivity” OR “specificity”)
3) TS=(“hyperparathyroidism” OR “parathyroid adenoma”)

1) AND 2) AND 3)
